# Obesity-Induced Insulin Resistance in Human Skeletal Muscle Is Characterised by Defective Activation of p42/p44 MAP Kinase

**DOI:** 10.1371/journal.pone.0056928

**Published:** 2013-02-28

**Authors:** Antonio J. Ruiz-Alcaraz, Christopher Lipina, John R. Petrie, Michael J. Murphy, Andrew D. Morris, Calum Sutherland, Daniel J. Cuthbertson

**Affiliations:** 1 Department of Biochemistry and Molecular Biology B and Immunology, School of Medicine, University of Murcia, Murcia, Spain; 2 Division of Cell Signalling and Immunology, University of Dundee, Dundee, United Kingdom; 3 Institute of Cardiovascular and Medical Sciences, University of Glasgow, Glasgow, United Kingdom; 4 Division of Cardiovascular and Diabetes Medicine, University of Dundee, Ninewells Hospital, Dundee, United Kingdom; 5 Department of Obesity and Endocrinology, Clinical Sciences Centre, University Hospital Aintree, Liverpool, United Kingdom; 6 Department of Obesity and Endocrinology, Institute of Aging and Chronic Disease, University of Liverpool, Liverpool, United Kingdom; University of Las Palmas de Gran Canaria, Spain

## Abstract

Insulin resistance (IR), an impaired cellular, tissue and whole body response to insulin, is a major pathophysiological defect of type 2 diabetes mellitus. Although IR is closely associated with obesity, the identity of the molecular defect(s) underlying obesity-induced IR in skeletal muscle remains controversial; reduced post-receptor signalling of the insulin receptor substrate 1 (IRS1) adaptor protein and downstream effectors such as protein kinase B (PKB) have previously been implicated. We examined expression and/or activation of a number of components of the insulin-signalling cascade in skeletal muscle of 22 healthy young men (with body mass index (BMI) range, 20–37 kg/m^2^). Whole body insulin sensitivity (M value) and body composition was determined by the hyperinsulinaemic (40 mU. min^−1^.m^−2^.), euglycaemic clamp and by dual energy X-ray absorptiometry (DEXA) respectively. Skeletal muscle (vastus lateralis) biopsies were taken before and after one hour of hyperinsulinaemia and the muscle insulin signalling proteins examined by western blot and immunoprecipitation assay. There was a strong inverse relationship between M-value and BMI. The most striking abnormality was significantly reduced insulin-induced activation of p42/44 MAP kinase, measured by specific assay, in the volunteers with poor insulin sensitivity. However, there was no relationship between individuals' BMI or M-value and protein expression/phosphorylation of IRS1, PKB, or p42/44 MAP kinase protein, under basal or hyperinsulinaemic conditions. In the few individuals with poor insulin sensitivity but preserved p42/44 MAP kinase activation, other signalling defects were evident. These findings implicate defective p42/44 MAP kinase signalling as a potential contributor to obesity-related IR in a non-diabetic population, although clearly multiple signalling defects underlie obesity associated IR.

## Introduction

Insulin resistance (IR) is a common pathophysiological state in which higher than normal concentrations of insulin are required to exert its biological effects in target tissues such as skeletal muscle, adipose tissue and liver [1]. It is frequently associated with a number of diseases including obesity, type 2 diabetes mellitus (T2DM), polycystic ovary syndrome (PCOS) [2] and non-alcoholic fatty liver disease (NAFLD) [3]. Skeletal muscle IR contributes significantly to the metabolic derangements seen in these patients considering that skeletal muscle accounts for the majority of insulin-mediated glucose disposal in the post-prandial state [4].

The molecular basis of skeletal muscle IR is assumed to be due to post-receptor defects in the insulin signal transduction pathway, culminating in impaired translocation of the glucose transporter GLUT4 to the cell membrane [5], [6]. Most evidence implicates functional defects such as impaired phosphorylation or activation, rather than aberrant protein expression but these observations mainly derive from studies in animals or analysis of relatively few human biopsies. Insulin signalling is complex although the majority of its control over glucose homeostasis requires two pathways downstream of the insulin receptor substrates (IRSs): namely the PI-3K/PKB pathway and the p42/p44 MAPK (ERK 1/2) pathway. There is evidence for increased phosphorylation of a number of serine/threonine (Ser/Thr) sites on IRS1 in human muscle with a subsequent impairment of tyrosine phosphorylation of IRS1, thereby reducing downstream activation of PI-3 kinase and PKB and hence decreasing activation of glucose transport and other downstream events [5], [7]–[9]. Similarly in obesity, in intact muscle strips, there is impaired IRS-1 tyrosine phosphorylation and PI-3 kinase activity in response to insulin stimulation [10].

We have previously demonstrated, in human skeletal muscle from healthy controls, that IRS1 protein expression levels are actually increased 3-fold following 1 h of hyperinsulinaemia. Hence impairment of this induction of IRS1 would reduce downstream insulin signalling capacity, thereby contributing to the development of IR prior to the appearance of T2DM [11]. Indeed, in mice, mutation of the serine 307 residue (Ser307) to alanine (Ala) in IRS1, rendering IRS1 incapable of being phosphorylated, reduced the insulin sensitivity of these animals, suggesting a key role for Ser307 of IRS1 in regulating insulin sensitivity [12]. Meanwhile, a recent global analysis of IRS1 phosphorylation indicated that multiple residues on IRS1 exhibit altered phosphorylation in T2DM muscle but that these are not abnormal in muscle from a pre-diabetic obese group [13]. This questions the role of IRS1 phosphorylation in the initial development of IR in this population. Interestingly induction of Ser/Thr phosphorylation of IRS1 by insulin was similar in both the control and T2DM volunteers, suggesting that obesity-induced IR did not affect this aspect of insulin action. We have previously reported that dysregulation of p42/p44 MAPK, rather than other signalling proteins, may underlie reduced skeletal muscle glucose transport and development of IR in ageing and in PCOS [2], [14]. Therefore, it remains unclear whether all cases of IR with obesity have defective PI3K-PKB signalling in the muscle and whether additional signalling defects contribute to progression to T2DM.

The main aims of the current study were to establish whether defective insulin signalling could be detected in human skeletal muscle correlating with insulin resistance prior to the development of diabetes, and determine whether this consisted of a single common defect, or a range of signalling defects.

## Materials and Methods

### Ethics Statement

The subjects were informed of the experimental protocol both verbally and in writing before giving their informed consent. The experimental protocol was approved by the Tayside Ethics Committee and was carried out according to the Helsinki Declaration.

### Participant characteristics

Twenty two healthy men, 29±8y (SEM) participated in the study. None were taking regular medication. Height (metres) and weight (kilograms) were measured to determine the body mass index (BMI). The cohort was recruited to include four groups of individuals: seven of normal weight (BMI, 20–24 kg/m^2^), five overweight (BMI 25–29) and ten obese (BMI≥30 kg/m^2^). The subjects were not habitually active. They were instructed to adhere to their usual diet and to refrain from any strenuous physical activity for 2 days before the study.

### Study protocol

All subjects had a screening visit to assess family history of diabetes and to confirm normal glucose tolerance with a standard (75 g of powdered glucose) 2-hour oral glucose tolerance test. Those individuals with a first-degree relative known to have diabetes or with diabetes according to the World Health Organisation diagnostic criteria (fasting glucose ≥7.0 mmol/l or 2 h glucose ≥11.1 mmol/l) were excluded from the study. Body composition (fat mass, fat free mass and bone mineral content) was determined by dual energy photon X-ray absorptiometry (DEXA; HOLOGIC Discovery W, Bedford, MA, version 12.1).

Eligible subjects attended the Clinical Investigation Unit, Ninewells Hospital at 08:00 having not eaten for 12 hours, for a study protocol involving skeletal muscle biopsies and a hyperinsulinaemic, euglycaemic clamp [15]. A cannula was introduced into a forearm vein for infusion of insulin (Actrapid, NovoNordisk Copenhagen, Denmark) and 20% dextrose while on the contralateral side a wrist or hand vein was cannulated retrogradely for blood sampling. This arm was heated in a hot box at 65°C throughout.

Quadriceps (vastus lateralis) muscle biopsies were taken, using 1% lignocaine anesthetic and the conchotome technique [16] at the start of the hyperinsulinaemic, euglycaemic clamp. All biopsies were taken using separate incisions and made from distal to proximal areas of the quadriceps. The biopsy was snap-frozen in liquid nitrogen and stored at −80°C until further analysis. Insulin was infused at 40 mU. min^−1^.m^−2^ body surface area followed by a variable infusion of 20% glucose to maintain plasma glucose concentration at 5.2 mmol/l. A second muscle biopsy was taken through a separate incision after one hour of the clamp. The clamp was maintained for a further hour to assess insulin sensitivity (M-value, see below).

### Analytical Methods

#### Plasma

Plasma was separated from whole blood by centrifugation (300 *g*) immediately after collection. Plasma glucose was measured with a YSI Stat2300 (Yellow Spring Instruments, Yellow Spring, OH) immediately after collection of each sample; the remainder of the plasma sample was frozen until further analysis. Plasma insulin was measured by the Clinical Biochemistry Department at Ninewells Hospital, Dundee, using a Siemens Immulite 2000 Immunoassay system.

#### Preparation of Protein Extracts for Western Blotting or Immunoprecipitation

Protein extracts were obtained as detailed previously[11]. In brief, muscle biopsies were thawed in ice and homogenized (Dounce, 10–15 strokes) in 0.5 ml ice-cold lysis buffer (25 mM Tris-HCl (pH 7.4), 50 mM NaF, 100 mM NaCl, 1 mM sodium vanadate, 5 mM EGTA, 1 mM EDTA, 1% (v/v) Triton X-100, 10 mM sodium pyrophosphate, 0.27 M sucrose, Complete Protease inhibitor cocktail tablets (1 tablet/10 ml), and 0.1% (v/v) 2-mercaptoethanol). Protein lysates were obtained from the supernatant fraction after 10 min centrifugation at 13,000 r.p.m., and then pre-cleared for 1 h at 4°C with Protein G-Sepharose in PBS 50% (v/v). This process removed contaminating antibodies present in the samples due to excess blood in the muscle samples. We found this step to be vital for consistent results with the subsequent immunoblot and immunoprecipitation steps.

#### Western Blot Analysis

Protein extracts (30–40 µg protein) were separated on Novex SDS 4–12% polyacrylamide gels. Following transfer to nitrocellulose membranes, blots were first blocked with 5% (w/v) non-fat milk in TBST (Tris-buffered saline containing 0.1% (v/v) Tween 20) for 1 h, and incubated with primary antibodies at 4°C overnight prior to incubation for 1 h at room temperature with the secondary antibody and, finally development was carried out using an enhanced chemiluminescence (ECL) kit (Amersham Biosciences, Inc.). Bands obtained were quantified by densitometry using Scion Image software. Pre- and post-insulin protein lysates from each individual were run on gels as pairs, with 4 volunteers per gel, along with a gel-to-gel normalisation control (volunteer 14, BMI of 24). Densitometry readings for every volunteer could then be normalised to volunteer 14 for comparison of either basal or post-insulin data. However this normalisation was not required when assessing response to insulin as this was done within a single immunoblot, after normalisation to the appropriate loading control (phosphorylation normalised to expression of same protein but on a separate gel using same sample run at same time rather than following stripping, while protein expression normalised to β-actin). To ensure that individual biopsies contained an equal amount of muscle protein and were not contaminated by variable amounts of adipose tissue, the relative levels of Troponin C (muscle marker) and Fatty Acid binding protein-4 (adipose enriched protein also called aP-2) were measured by immunoblot. The ratio of these proteins did not vary by more than 20% across the entire 44 biopsies, and did not correlate with BMI or M-value (data not shown).

#### Immunoprecipitation and kinase assays

Protein extracts (50–250 µg) were incubated for 1 h on a shaking platform with protein G-sepharose conjugated to 2 mcg of anti-PKB antibody (PH domain), Upstate (Lake Placid, USA) or anti-p42/p44 MAP kinase antibody from Abcam (Cambridge, UK). The immunocomplexes were pelleted and washed with 1 ml of Lysis Buffer containing 0.5 M NaCl, and twice with 1 ml of assay buffer (25 mM MOPS pH 7.0, 0.4 mM EDTA, 0.1 M NaCl, 0.01% Brij35 and 0.1% (v/v) 2-mercaptoethanol). The immunoprecipitated kinases were incubated at 30°C for 30 min, in a total volume of 50 μcl containing 25 mM MOPS (pH 7.0), 0.4 mM EDTA, 0.1 M NaCl, 0.01% Brij35, 0.1% (v/v) 2-mercaptoethanol, 10 mM MgCl, 0.1 mM [γ-32P]ATP (0.5×106 c.p.m./nmol) and 30 μicromolar Crosstide (GRPRTSSFAEG) or 0.1 mM MBP as respective substrates to PKB and ERK. A Unit of kinase activity is defined as the amount which catalyses the phosphorylation of 1 nmol of substrate in 1 hour.

#### Insulin Sensitivity Calculations

Whole body insulin sensitivity was assessed by determining the amount of glucose metabolised (M value; mg.kg^−1^.min^−1^) according to the infusion rate of exogenous glucose and using the space correction to account for under or overfilling the glucose space, with blood glucose concentrations recorded every five minutes during the final 20-minute period. Values are expressed per kilogram of total body weight.

#### Antibodies

Rabbit polyclonal antibody to IRS1 (raised against 14 C-terminal aminoacids) and a mouse monoclonal PKB antibody (raised against the PH domain) were purchased from Upstate Biotechnology (Lake Placid, NY, USA), the antibodies to ERK1/2, FABP-4 and Troponin C were obtained from Abcam (Cambridge, UK), and the antibody to β-actin was purchased from Sigma-Aldrich (St Louis, MO, USA). Antibodies against phospho-PKB (Ser473) and (T308), phospho-p42/p44 MAP kinase (Thr202/Tyr204), phospho-FOXO1 (Ser256), phospho-GSK-3 ˜αβ (Ser9/21), GSK3-β and phospho-S6 ribosomal protein (Ser240/244) were purchased from Cell Signalling Technology (Danvers, MA, USA). The Division of Signal Transduction and Therapy, University of Dundee generated the antibodies to FOXO1 and p70S6 kinase in-house in sheep.

### Statistical analysis

Correlation was performed using Pearson correlation for normally distributed variables and using Spearman correlation for variables not normally distributed.

## Results

### Biochemistry

Mean (±SD) fasting plasma glucose concentrations were all within the normal concentration range at 5.1±0.6 mmol/l. Serum insulin concentrations increased from a mean (±SD) fasting concentration of 5±3 µU/ml (n = 11) to 62±17 µU/ml after 1 h of hyperinsulinaemia (n = 7).

### Body composition

Of the 22 individuals, 7 were of normal weight (BMI, 20–24 kg/m^2^), 5 were overweight (BMI 25–29 kg/m^2^) and 10 were obese (BMI≥30 kg/m^2^). 19 out of 22 individuals underwent body composition analysis using whole body DEXA. There was a highly significant correlation between BMI and fat mass, measured by DEXA (r = 0.85; p<0.001).

### Relationship between body composition and insulin sensitivity

**Figure 1:**
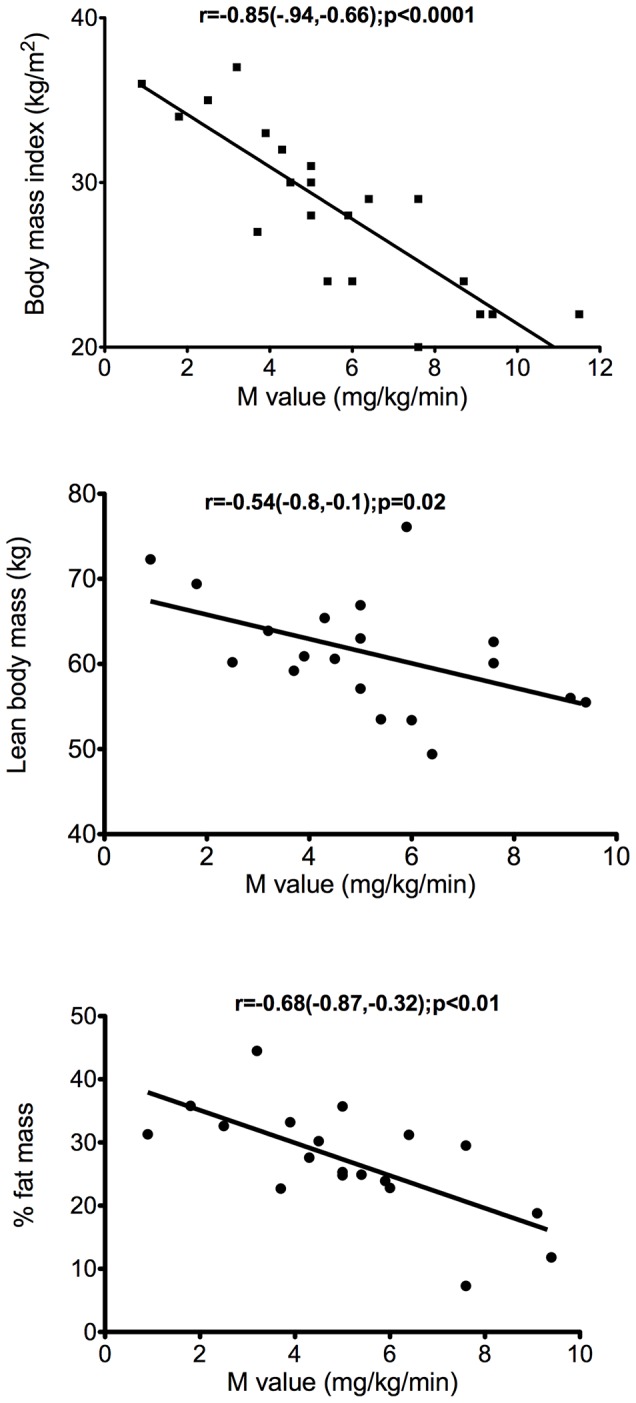
Relationship between whole body insulin sensitivity and body mass index, lean body mass and percentage fat mass. Whole body insulin sensitivity (M value; mg/kg/min) and A) body mass index (kg/m^2^), B) lean body mass (kg) and C) percentage fat mass.

There was a highly significant inverse correlation between the whole body insulin sensitivity, M value and BMI (r = −0.85; p<0.0001), lean body mass (r = −0.8; p = 0.02) and percentage fat mass (r = −.68; p = 0.01) (Figure 1), confirming that the lean patients were insulin-sensitive and that there was an increasing degree of IR relative to increasing obesity.

### Protein expression

#### PKB and p42/44 MAPK

There was inter-individual variability in expression of PKB and p42/44 MAPK both in the fasted or post 1 h insulin biopsies, with no correlation of each to either BMI or whole body insulin sensitivity (M-value) (data not shown).

#### IRS1

**Figure 2:**
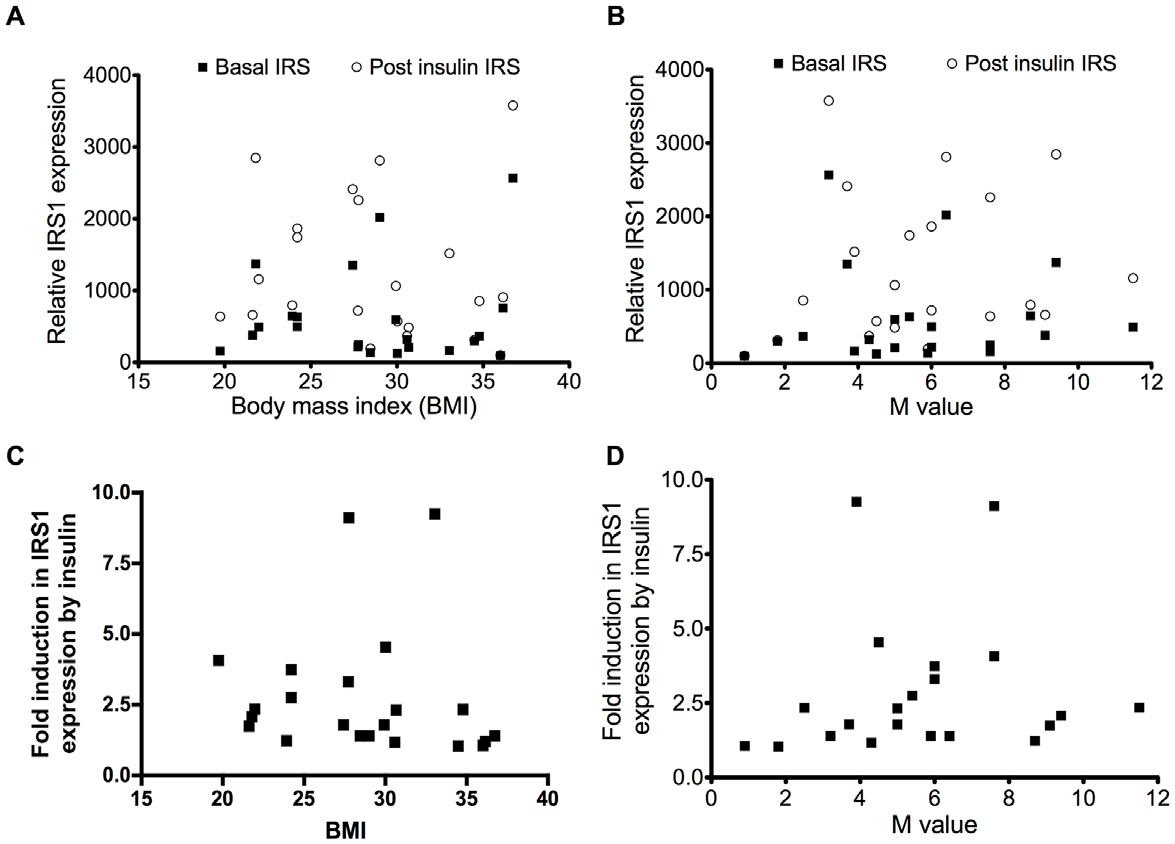
Relationship of IRS1 expression with body mass index or M value. Relative IRS1 protein expression according to body mass index (A) or to M value (B) and fold increase in IRS1 expression according to body mass index (r = −0.36; p = 0.10) (C) or to M value (r = 0.27; p = 0.23) (D).

Again, there was a great deal of inter-individual variation in the IRS1 levels in the fasted state. IRS1 expression in muscle increases following acute exposure to insulin (Figure 2, A and B); there was no significant correlation between fold induction of IRS1 protein expression in response to insulin and the BMI (r = −0.36; p = 0.10), or M value (r = 0.27; p = 0.23) (Figure 2, C and D).

### Phosphorylation status

#### PKB

**Figure 3:**
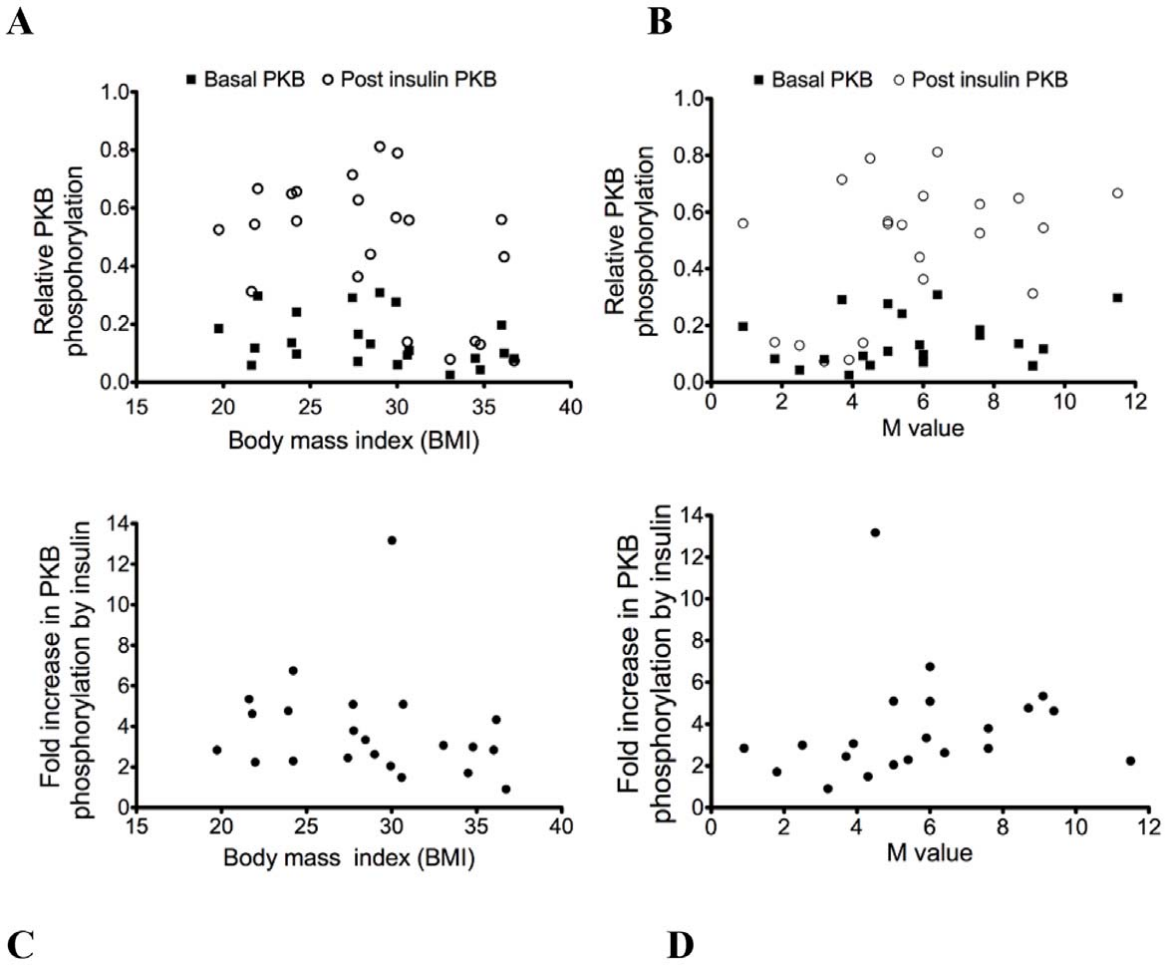
Relationship of PKB phosphorylation with body mass index or M value. Relative PKB phosphorylation according to body mass index (A) or to M value (B) and fold increase in PKB phosphorylation by insulin according to body mass index (r = −.38; p = 0.09) (C) or to M value (r = 0.4; p = 0.08) (D).

The induction of PKB phosphorylation by insulin was apparent in most volunteers (Figure 3 A and B). There was a tendency for the degree of insulin-induced phosphorylation of PKB to reduce with increasing BMI (r = −.38; p = 0.09) (C) and to increase with increasing M value (r = 0.4; p = 0.08) (D) but these failed to reach significance. In contrast to the analysis of p42/p44 MAPK, direct assay of PKB activity rather than western blotting of phosphorylation failed to improve the correlation between PKB activity and insulin sensitivity (data not shown).

#### p42/44 MAPK

**Figure 4:**
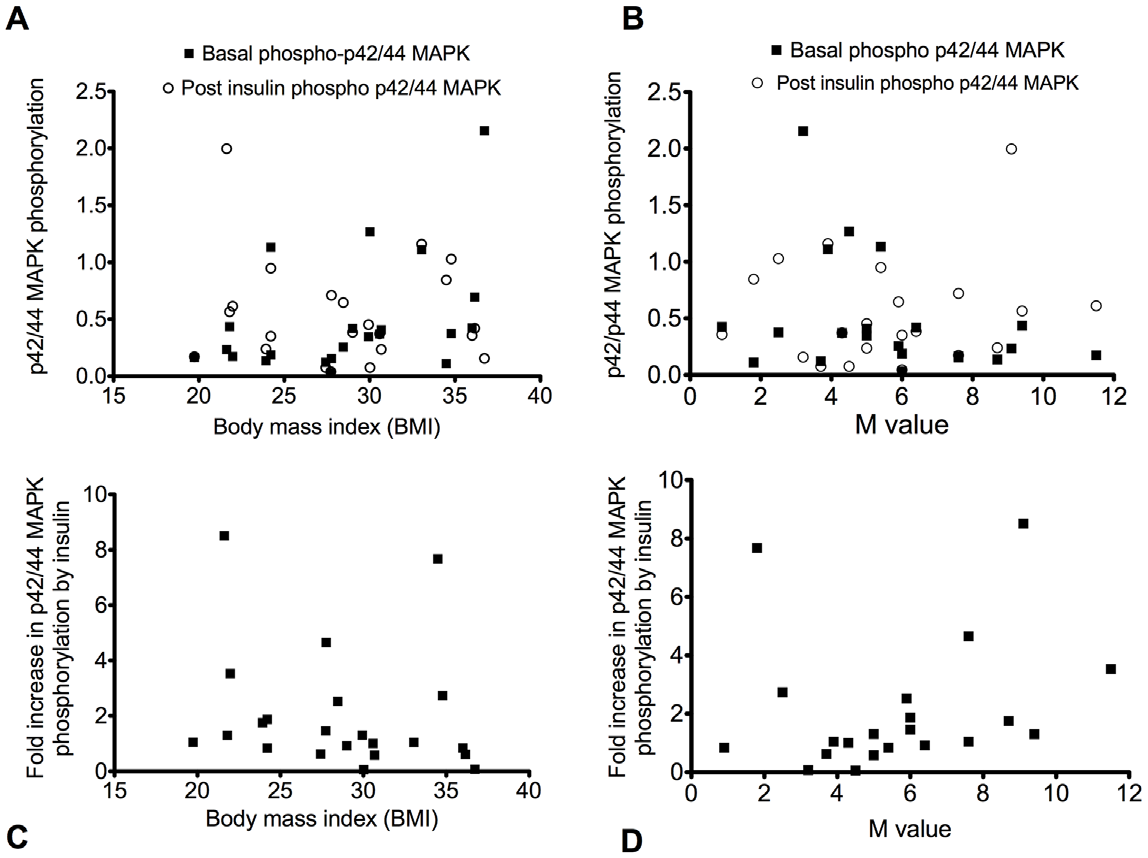
Relationship of ERK phosphorylation with body mass index or M value. Relative ERK phosphorylation according to body mass index (A) or to M value (B) and fold increase in ERK phosphorylation by insulin according to body mass index (r = 0.4; p = 0.07) (C) or to M value (r = 0.59; p = 0.08) (D).

There were no significant correlations between basal p42/44 MAPK phosphorylation and either BMI or M value (Figure 4). There was a tendency for p42/44 MAPK phosphorylation following insulin exposure to correlate with BMI (Spearman r = 0.4; p = 0.07) (C) or with M value (Spearman r = 0.59; p = 0.08) (D) but these both failed to reach significance.

### p42/44 MAPK Activity

**Figure 5:**
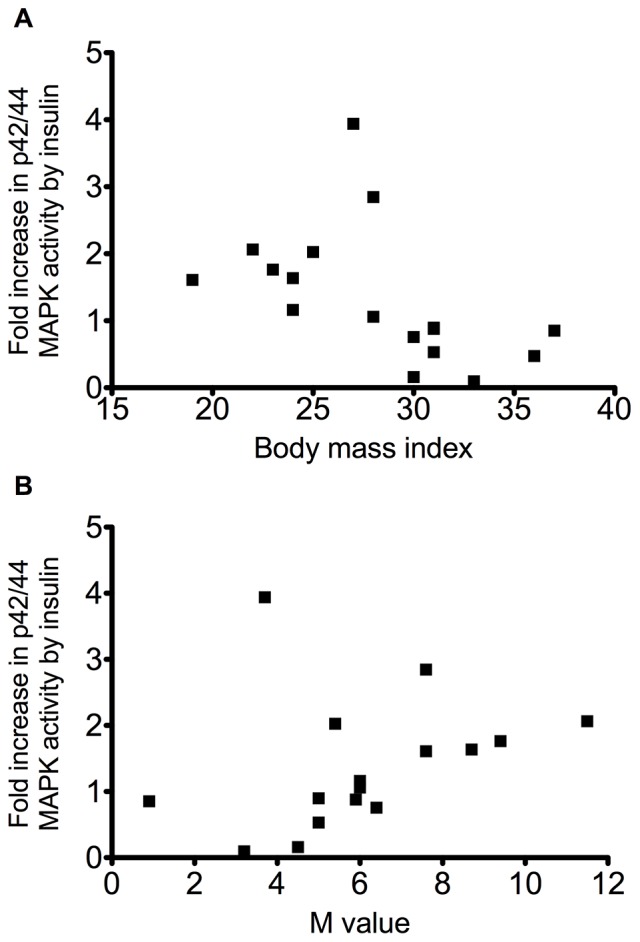
Fold activation of ERK by insulin according to body mass index or M value. (A) Body mass index (r = 0.73; p = 0.0009) or (B) M value (r = 0.52; p = 0.04).

It is widely appreciated that the western blot technique is semi-quantitative (albeit technically relatively straightforward). Therefore, since there was an indication that insulin regulation of p42/44 MAPK phosphorylation may be reduced in the more insulin resistant population, we decided to perform a more quantitative analysis of p42/44 MAPK activation. p42 MAPK and p44 MAPK were immunoprecipitated prior to an *in vitro* assay of activity, for the pre- and post-insulin biopsies, for 17 of the 22 volunteers, covering the full range of BMI and M-values. As with western blot we found that there was a great deal of inter-individual variability in the basal level of activity. There was no significant correlation between either basal activity or post-insulin p42/p44 MAPK activity levels, and M-value or BMI (Figure 5). However there was an inverse correlation between fold-induction of p42/44 MAPK activity by insulin and body mass index (r = 0.73; p = 0.0009) (Figure 5A) and a significant correlation between p42/44 MAPK activity in response to insulin and M value (r = 0.52; p = 0.04) (Figure 5B). Thus, whether measured against the degree of obesity or IR, the data indicates a close relationship between defective response to insulin of p42/44 MAPK activity in muscle and the clinical measures of pre-diabetes. This suggests that abnormal p42/p44 MAPK response to insulin in skeletal muscle is a better marker of whole body insulin resistance than the response of the PI3K-PKB pathway, at least in obese non-diabetic individuals.

#### FOXO, GSK3 and ribosomal S6

There were no correlations between the basal or insulin-induced levels of phosphorylation of FOXO, GSK3 and ribosomal S6 protein with either BMI or M value (data not shown).

### Summary of signalling analysis (Table 1)

**Figure 6:**
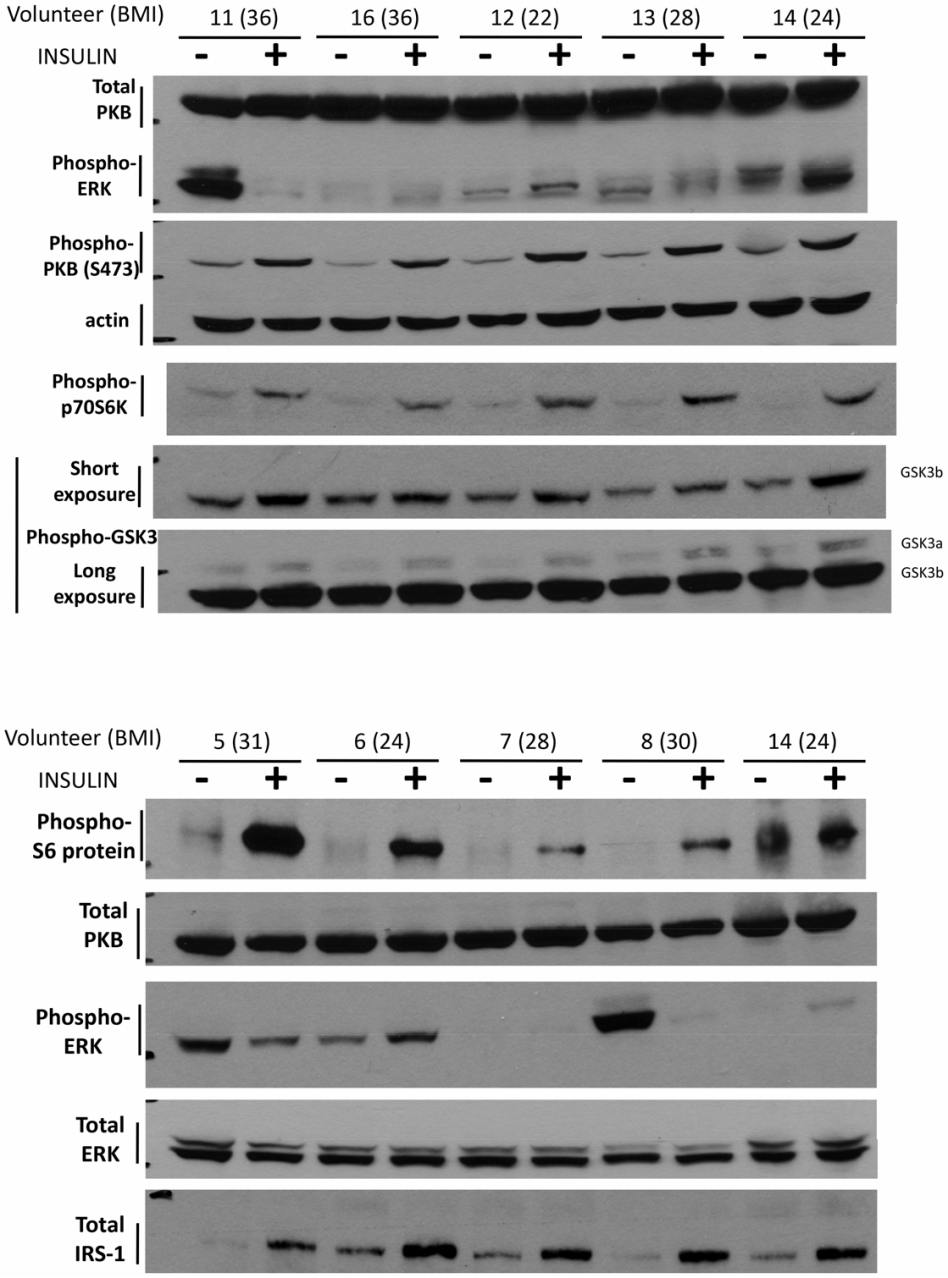
Representative Western blots. Body mass indices (BMI) are shown in parentheses and effects of fasting (−) or insulin (+).

The study group was stratified incrementally according to their whole body insulin resistance, determined by the M value, and the responses of each individual signalling protein to insulin were ranked and the four individuals with the greatest (Green numbers, ranking 1 to 4)) or least (Red numbers, ranking 1 to 4) responses for each protein were noted. Representative blots are shown (Figure 6). The responses of interest were insulin-induced changes in IRS1 protein expression, in PKB or p42/p44 MAP kinase phosphorylation or in p42/p44 MAP kinase activity.

We observed a general clustering of the greatest signalling responses to insulin within the more insulin sensitive individuals (those with higher M values who tended to have lower BMIs) and conversely the lowest signalling responses occur in the more insulin resistant individuals (those with the lower M values who tended to have higher BMIs). However, defects in multiple signalling proteins often co-existed in the same individual but moreover no single signalling defect explained insulin resistance in all obese individuals.

## Discussion

The main findings of the current study implicate, for the first time, attenuation of insulin stimulation of the p42/p44 MAP kinase pathway as an early defect in obesity-induced IR in skeletal muscle. We observed an impaired insulin-induced stimulation of skeletal muscle p42/p44 MAP kinase activity, in those individuals with higher BMI or lower whole body insulin sensitivity (M value) i.e. obese, IR individuals. Consistent with this finding, in the same individuals, we observed blunting of insulin-induced phosphorylation of p42/p44 MAP kinase at the regulatory residues (Tyr185, Thr183). Importantly, this signalling defect only became apparent when assessing the magnitude of response to insulin, and was not detectable by measuring p42/p44 MAP kinase activity or phosphorylation, in the post-absorptive state, without insulin stimulation. Our findings also suggest that multiple molecular mechanisms may mediate skeletal muscle IR within different overweight or obese individuals. Although defective p42/p44 MAP kinase signalling was the most prevalent in the volunteers studied there was clear evidence of defects in other signalling proteins including IRS1 and PKB in several individuals.

These observations are entirely consistent with previous analyses from this group demonstrating an association of p42/p44 MAP kinase signalling with stimulation of muscle glucose transport [17] as well as a defective regulation of the p42/p44 MAP kinase pathway in different pathophysiological conditions associated with skeletal muscle IR and reduced glucose transport. First, in young women with PCOS, there was a severe attenuation of insulin stimulation of the p42/p44 MAP kinase pathway in muscle compared to controls (with p42/p44 MAP kinase activity actually reducing in response to insulin) although the group was too small to detect any correlation between BMI and the defective regulation of p42/p44 MAP kinase in the PCOS group [2]. Others have reported that the p42/p44 MAP kinase pathway is constitutively activated both in vitro and in vivo in the skeletal muscle of women with PCOS [18]. Similarly, in older healthy subjects and in patients with T2DM, in whom skeletal muscle uptake of 2-deoxyglucose was blunted compared with healthy young men, reduced stimulation of p42/44 MAP kinase phosphorylation was observed [14]. In contrast, Cusi *et al* reported that insulin stimulation of the p42/p44 MAP kinase was normal in obese and diabetic subjects [19]. Furthermore, Jager *et al* demonstrated that specific inactivation of p44 MAP kinase in obese, leptin deficient mice protected them against insulin resistance despite massive obesity. These animals exhibited relatively good whole-body insulin sensitivity and increased insulin action in skeletal muscle compared to control animals [20]. However all of the studies suggest that this pathway exerts control over insulin action, where chronic deletion may generate compensatory insulin sensitising mechanisms but the initial loss of insulin induction of p42/p44 MAP kinase may be a marker of defective insulin action in muscle in response to obesity.

Others have suggested that defective IRS1 or IRS2 signalling is present in muscle of patients with T2DM. Supporting this hypothesis, a genetic variant near IRS1, that is associated with reduced basal levels of IRS1 protein and decreased insulin induction of IRS1-associated PI-3K activity in human skeletal muscle biopsies, is associated with type 2 diabetes, insulin resistance and hyperinsulinemia [21]. We have previously reported a significant increase in IRS1 protein expression following acute insulin treatment of human muscle [11]; however the fold induction of IRS1 expression in response to insulin in this study was not correlated with either BMI or M value. We cannot rule out abnormalities in one or more of the many post translational modifications of this protein (or its homologue IRS2), however we have focussed on distal signalling mechanisms where deficits in IRS1 function would still be detectable. For example, a mutation in PKB beta has been found to associate with severe IR and lipodystrophy, demonstrating the importance of the IRS-PI3K-PKB pathway to insulin sensitivity [22], although mutations in this protein appear to contribute to only a very small fraction of IR in the population [23]. Our data suggest that there are relatively few cases of defective IRS1-PKB signalling that correlate with obesity induced insulin resistance in an otherwise healthy population. We measured protein expression, the phosphorylation (at a residue known to regulate activity in response to insulin) and where possible the inherent activity of PKB, p42/p44 MAPK GSK3, FOXO1 and p70S6K. Although we could not detect abnormalities in PKB activation by insulin, there was an indication that the phosphorylation of PKB at Ser473 may be higher in the muscle of the more insulin sensitive group, at least after exposure to insulin, although the differences were not significant. Indeed, the dissociation of whole body IR from defects in proximal insulin signaling in obese volunteers that we observe are also consistent with those observations of several other groups [24], [25]. GSK3 and FOXO are more distal downstream targets of PKB while S6 protein is regulated by the mTOR pathway [26]. No significant differences were observed in basal or insulin-induced phosphorylation of these molecules. This provides added confidence that the PI-3K pathway is responding to insulin in a similar fashion across the population studied.

Despite the correlation of defective p42/p44 MAP kinase activation and poor insulin sensitivity there were still some obese, insulin resistant individuals in whom p42/p44 MAP kinase could readily be activated in response to insulin (Fig.4). However, in all of the most insulin resistant subjects at least one major signalling defect in their muscle was evident, when assessed as response to insulin (*but not when examined as activity or expression of a signalling molecule in the post-absorptive state*). However, the individuals with the greatest induction of these molecules tended to have lower BMI and higher M-values, and conversely the subjects with poorest responses to insulin at the molecular level generally also had low M-values and high BMI scores (Table 1). It is worth noting that the 10 individuals with lowest whole body insulin sensitivity had complete loss of insulin induction of at least one of the major signalling molecules. However it was not always the same molecular defect: for example in subjects 15, 5 and 8, p42/p44 MAP kinase activity was suppressed (instead of increased) in response to insulin, while subject 22 exhibited no insulin induction of PKB phosphorylation or IRS1 protein (despite strong induction of p42/p44 MAPK phosphorylation and activity). It is not immediately obvious why different signalling defects should arise in a relatively healthy obese population, however it may be related to dietary variations with different compositions of fatty acids altering signalling in different ways [27], or other lifestyle factors not apparent in our study. This aspect, as well as establishing whether one signalling defect is more liable to promote diabetes, deserves further investigation.

**Table 1:** Summary table.

BMI	M-value	IRS1 proteinexpression	Change in PKB phosphorylation	p42/44 MAP kinasephosphorylation	p42/44 MAP kinase activity
36	0.9	L2			L4
35	1.8	L1	L3	H2	
35	2.5				
37	3.2	L4	L1	L2	L1
27	3.7			L4	H1
33	3.9	H1			
31	4.3	L3	L2		
30	4.5	H3	H3	L1	L2
30	5		L4		
31	5			L3	L3
24	5.4				H4
29	5.9				
24	6		H1		
28	6		H4		
29	6.4				
28	7.6	H2		H3	H2
20	7.6	H4			
24	8.7				
22	9.1		H2	H1	
22	9.4				
22	11.5			H4	H3

The study group is presented from the lower to the higher M-value, demonstrating clustering of signalling abnormalities stratified in ascending order according to the induction of signalling changes in response to insulin. Least potent induction is ranked as lowest L1 to L4; most potent induction is ranked as highest H1 to H4.

In summary, aberrant p42/p44 MAPK signalling was the most common problem found in obesity-induced insulin resistant skeletal muscle. However, multiple defects in insulin signal transduction were apparent in this group and it will be of interest to establish whether the p42/p44 MAPK defect is associated with progression to T2DM.
